# WHAT CAN WE LEARN FROM THE THREE MURDER CASE IN A FEE-BASED NURSING HOME IN JAPAN?

**DOI:** 10.1093/geroni/igac059.1122

**Published:** 2022-12-20

**Authors:** Noriko Tsukada, Asako Katsumata

**Affiliations:** Nihon University, Tokyo, Tokyo, Japan; Rehabilitation Institution, Chiba, Chiba, Japan

## Abstract

Japan enacted “The Act on the Prevention of Elder Abuse, Support for Caregivers of Elderly Persons and Other Related Matters” on 2006 April 1. Although the Act enabled the implementation of new programs and procedures to address abuse, elder abuse remains a cause of concern, particularly in institutional settings. This paper presents an elder abuse case that occurred in 2014 in a fee-based nursing home where a staff member murdered three residents. Although extreme, we chose to present this case because there is minimal research focused on fee-based nursing homes. Moreover, this case provides an opportunity to look broadly at factors that need to be addressed to reduce the likelihood of elder abuse in institutional settings. This paper first introduces facts about this case and discusses what can be learned to help increase an understanding about preventing future elder abuse cases in institutional settings.

